# MicroRNAs in the Cholangiopathies: Pathogenesis, Diagnosis, and Treatment

**DOI:** 10.3390/jcm4091688

**Published:** 2015-08-26

**Authors:** Maria Jose Lorenzo Pisarello, Lorena Loarca, Tommy Ivanics, Leslie Morton, Nicholas LaRusso

**Affiliations:** 1 Division of Gastroenterology and Hepatology, and the Mayo Clinic Center for Signaling in Gastroenterology, Mayo Clinic, Rochester, MN 55905, USA; E-Mails: Pisarello.maria@mayo.edu (M.J.L.P.); Loarca.Lorena@mayo.edu (L.L.); Morton.Leslie@mayo.edu (L.M.); 2 Department of Surgery, Mayo Clinic Rochester, MN 55905, USA; E-Mail: Ivanics.Tommy@mayo.edu

**Keywords:** microRNA, cholangiopathies, cholangiocarcinoma

## Abstract

The cholangiopathies are a group of liver diseases resulting from different etiologies but with the cholangiocyte as the primary target. As a group, the cholangiopathies result in significant morbidity and mortality and represent one of the main indications for liver transplant in both children and adults. Contributing to this situation is the absence of a thorough understanding of their pathogenesis and a lack of adequate diagnostic and prognostic biomarkers. MicroRNAs are small non-coding RNAs that modify gene expression post-transcriptionally. They have been implicated in the pathogenesis of many diseases, including the cholangiopathies. Thus, in this review we provide an overview of the literature on miRNAs in the cholangiopathies and discuss future research directions.

## 1. Introduction

The biliary tree is a complex network of conduits that start at the canals of Hering and progressively merge into a system of intralobular, septal, and major intrahepatic ducts ultimately coalescing to form the extrahepatic bile ducts, which deliver bile to the gallbladder and the intestine. Area (zonal) and segmental ducts are considered large intrahepatic bile ducts, whereas septal ducts represent the transition point between the large and the interlobular biliary system. This classification is based on the diameter of the ducts: bile ductules (15 μm), interlobular ducts (15–100 μm), septal ducts (100–300 μm), area (zonal) ducts (300–400 μm), segmental ducts (400–800 μm), and hepatic ducts (800 μm) [1,2,3]. Small bile ducts are lined by 4 to 5 cuboidal cholangiocytes while larger bile ducts are lined by 10–12 cholangiocytes. The lining by the cholangiocytes of the latter is larger and more columnar in shape than the former [3,4,5].

Cholangiocytes possess an apical and basolateral plasma membrane. Extending from the apical membrane is a single primary cilium. Cilia are microtubule-based organelles that function as mechano-, chemo-, and osmosensors [6,7,8,9,10].

The major physiologic function of cholangiocytes is ductal bile formation which occurs through modification of primary bile secreted by hepatocytes through a series of secretory and reabsorptive processes. In addition, cholangiocytes are involved in regenerative/reparative processes.

**Figure 1:**
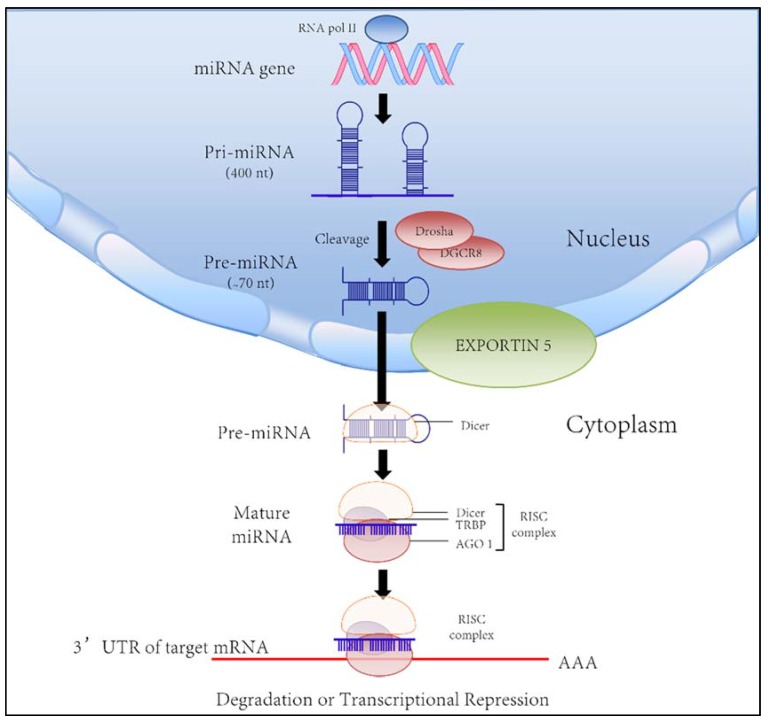
*microRNA biogenesis*. MicroRNA (miRNA) genes are transcribed by RNA polymerase II (Pol II) to generate the primary transcripts (pri-miRNAs). The initial processing of the primary transcript is mediated by the Drosha-DiGeorge syndrome critical region gene 8 (DGCR8; Pasha in Drosophila melanogaster and Caenorhabditis elegans) complex (also known as the Microprocessor complex) that generates ~70 nucleotide (nt) pre-miRNAs. Pre-miRNA has a short stem plus a ~2-nt 3′ overhang, which is recognized by the nuclear export factor exportin 5. Once exported from the nucleus, the cytoplasmic RNase III Dicer catalyses the production of miRNA duplexes. Dicer, TRBP (TAR RNA-binding protein; also known as TARBP2), and Argonaute (AGO) 1–4 mediate the processing of pre-miRNA and the assembly of the RISC (RNA-induced silencing complex). Within this complex, one strand of the miRNA duplex is removed resulting in a single stranded miRNA, partially complementary to target mRNA, which remains in the complex. .The “seed” sequence (positions 2–7 from the 5′ end of miRNAs) is complementary to the 3′ UTR of mRNA targets. microRNA complex interaction with mRNA induces posttranscriptional silencing through as both mRNA destabilization and translational repression (author’s figure) [14,15,16,17,18,19].

The cholangiopathies are diseases originating from the biliary tree, with biliary epithelial cells currently considered the primary cellular targets in their pathogenesis. These diseases include neonatal biliary atresia (BA), the main indication for liver transplantation in children. Indeed, about a third of all liver transplants in adults involve the cholangiopathies, primarily primary biliary cirrhosis (PBC) and primary sclerosing cholangitis (PSC) [11]. The cholangiopathies can be classified into several broad categories: immune-mediated, drug- or toxin-induced, infectious, genetic, ischemic, and idiopathic. Many of them evolve from chronic inflammation of the bile ducts, which subsequently leads to the development of cholestasis, bile duct proliferation and/or ductopenia (Table 1). This process can progress to biliary fibrosis and malignant transformation [12,13]. The pathogenic mechanisms involved in the cholangiopathies remain unknown; however, there is increasing evidence suggesting a role for miRNAs in the etiopathogenesis of at least some of the cholangiopathies. As a result, miRNAs have been postulated as potential targets for diagnosis and therapy. First described in 1993, miRNAs are a type of highly conserved, small non-coding RNAs with an average length of 22 nucleotides [14]. The biogenesis of miRNAs is shown in Figure 1.

**Table 1:** Classification of Cholangiopathies.

**Genetic**
Alagille’s syndrome
Cystic fibrosis
Fibropolycystic diseases (*i.e*., Caroli’s syndrome, congenital hepatic fibrosis, ADPKD, ARPKD, ADPLD)
**Immune-mediated**
Primary biliary cirrhosis
Primary sclerosing cholangitis
Hepatic allograft rejection
Graft *vs.* host disease involving the liver
Autoimmune cholangitis
**Infectious**
Bacterial cholangitis
Parasitic cholangitis
Fungal cholangitis
Viral cholangitis (*i.e.*, AIDS cholangiopathy)
Drug-induced (*i.e.*, Floxuridine-induced cholangiopathy)
Vascular/Ischemic (*i.e.*, postliver transplantation hepatic artery stenosis)
**Idiopathic**
Biliary atresia
Sarcoidosis Idiopathic childhood/adulthood ductopenia
**Malignant**
Cholangiocarcinoma (*i.e.*, bile duct adenocarcinoma)

ADPKD, Adult dominant polycystic kidney disease; ARPKD, Adult recessive polycystic kidney disease; ADPLD, Adult dominant polycystic liver disease; Adapted from Lazaridis *et al.*, 2004 [13].

Many key cellular processes involve regulation by miRNAs including cellular development, proliferation, apoptosis, metabolism, and morphogenesis [15,16,17]. Recently, aberrant miRNA expression has also been linked to disease. As a consequence, differentially expressed miRNAs in diseased tissues and circulating miRNAs in blood or other biological fluids may have a potential role in diagnosis and prognosis [18,19,20,21,22,23,24,25,26,27,28].

We previously published a review article highlighting the role of miRNAs in benign biliary tract diseases [29]. This review focuses on recent discoveries regarding miRNAs as biomarkers with respect to the cholangiopathies.

## 2. MicroRNAs in the Pathobiology of the Cholangiopathies

### 2.1. Biliary Atresia

Biliary atresia (BA) is a liver disease that occurs in infancy. It is characterized by inflammation, fibrosis, and obstruction of the extra-hepatic bile ducts (EHBD) [30].

**Figure 2:**
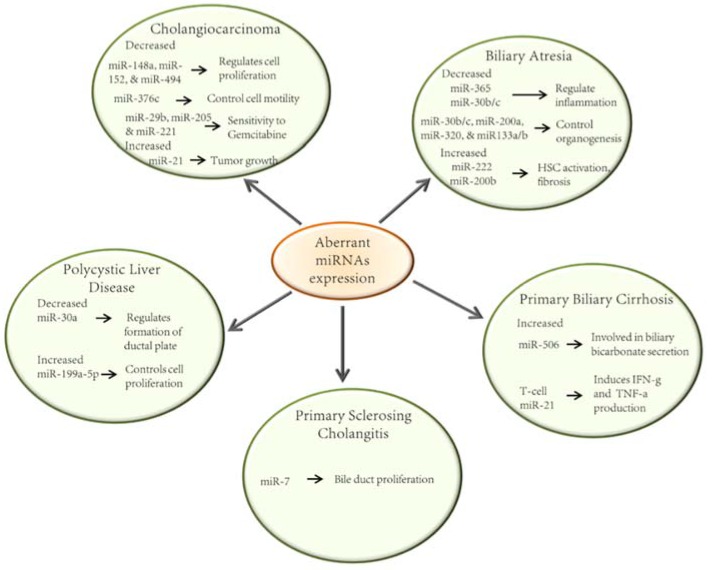
The roles of miRNAs on the pathogenesis of cholangiopathies. Schematic representation of the most relevant miRNAs whose expression is altered in cholangiophaties and their involvement in the pathogenesis of these diseases [31,33,34,35,37,38,39,40,41,42,43,44,45].

A number of miRNAs have been described to have altered expression in BA (Figure 2). These findings have been confirmed in both animals and humans. Bessho and colleagues have studied the biliary transcriptome in the Rhesus Rotavirus type-A (RRV) mouse model of BA. This model consists of three stages that occur within 14 days of RRV injection. Initially, biliary epithelial cells are injured (3 days), triggering an inflammatory response. This is followed by a blockade of bile duct lumens (7 days) and ultimately atresia of the bile duct (14 days). The group then investigated the presence of candidate miRNAs involved in bile duct obliteration during the development of BA. Their results demonstrated a 25.8-fold decrease in the expression of miR-let-7e in the RRV group compared to controls in the early stage of BA development (3 days following RRV challenge). Between 7 and 14 days of RRV exposure, the underexpression of at least 7 additional miRNAs was identified as potential candidates in the pathogenesis of BA. The levels of miR-30b/c, miR-133a/b, miR-200a, miR-320, and miR-365 were decreased by at least 2.36-fold compared to controls [31] Studies performed in embryonic mouse liver have demonstrated that miR-30b and miR-30c are induced at day 18.5 of embryogenesis and that miR-30c is necessary for ductal plate formation [31]. Furthermore, *in situ* hybridization studies of liver samples have shown miR-30c to be present in bile ducts of normal subjects. Moreover, miR-30c has been found to be upregulated in the ductal reaction that occurs in BA [32]. Importantly, miR-195, miR-365 and miR-30b/c regulate the expression of genes implicated in inflammation and miR-30b/c, miR-200a, miR-320 and miR-133a/b control organogenesis [31].

Aberrant miRNA levels have been suggested to play a crucial role in the development of liver fibrosis and hepatocellular carcinoma (HCC). In the RRV-mouse model of BA, miR-222 has been found to be increased in the EHBD. This increase has been shown to induce activation of hepatic stellate cells (HSC), which are important mediators of the fibrogenic response in BA [33]. The same group showed that miR-222 expression was considerably increased in liver tissue from BA patients compared to controls. A miR-222 binding site within the 3′UTR of the PPP2R2A gene was also demonstrated [33]. This gene is known to control cell proliferation through the Akt pathway. Additionally, abrogation of miR-222 in the human immortalized HSC cell line, LX-2, resulted in decreased cell proliferation via impairment of Akt signaling [34]. Almost in parallel, another group found that miR-200b promotes cell motility and replication in studies using the same cell line [35]. The mechanism involved activation of the PI3K/Akt pathway. Importantly, miR-200b was found to be overexpressed in liver specimens from children with BA-induced hepatic fibrosis. Furthermore, the expression of miR-200b increased with liver fibrosis progression [35]. Additionally, miR-21 has been implicated in the fibrogenesis of kidney, lung, and heart; and has been reported to be elevated in patients with BA [33]. Other miRNAs have been described to be associated with liver fibrosis in patients with BA. For instance, Roderburg and colleagues uncovered a link between miR-29 and the development of liver fibrosis [36]. In RRV-challenged mice, miR-29 is upregulated in hepatocytes and cholangiocytes. These changes in miR-29 expression result in decreased levels of insulin-like growth factor 1 (Igf1) and interleukin-1 receptor accessory protein (Il-1 RAP), two genes that have been implicated in the pathogenesis of BA [30].

### 2.2. Primary Biliary Cirrhosis

Primary biliary cirrhosis is a chronic autoimmune liver disease that primarily affects women. It is characterized by the presence of auto-reactive antibodies and T-cells, causing injury to the intrahepatic bile ducts and subsequently an uncontrolled fibrogenic response. This cascade of events can eventually lead to the development of liver cirrhosis [37].

Altered expression of miRNAs has also been associated with autoimmune disorders of the liver. Padgett and colleagues have reported the downregulation of miR-122a and miR-26a and the upregulation of miR-328 and miR-299-5p in liver tissue from patients with end-stage PBC. Importantly miR-122a represents 70% of all the miRNAs in the liver and is also involved in both NASH and HCC pathogenesis [46]. Another study identified elevated levels of miR-155 and miR-146a in peripheral blood mononuclear cells (PBMCs) in PBC patients compared to control patients. MiR-299-5p, also elevated in PBMCs of PBC patients, deemed resistant to ursodeoxycholic acid compared to control and treatment-responders [47].

A role of impaired biliary bicarbonate secretion has been suggested in the pathogenesis of PBC. Indeed, mice deficient in the Cl^−^/HCO_3_^−^ anion exchanger (AE2), a regulator of biliary bicarbonate flux, develop an autoimmune disease that resembles human PBC. Moreover, AE2 is decreased in PBMCs and in liver samples of patients with PBC compared to controls. MiR-506, was found to be upregulated in cholangiocytes in patients with PBC. It was also found that miR-506 binds to the 3′UTR of AE2 mRNA, blocking its translation. Interestingly, AE2 protein expression and function were restored when an anti-miR-506 was transfected into PBC cholangiocytes [37].

To further study PBC, an animal model was generated to express a dominant form of the TGF-β receptor type II (dnTGF-β RII). TGF-β signaling in this mouse is partially abrogated which leads to T-cell-induced autoimmunity, consequently, resulting in a cholangitic response similar to human PBC. Mice expressing dnTGF-β RII have increased levels of IL-17A, TNF-α and IL-6 in serum, colon, and liver *vs.* controls [38]. Furthermore, the T-cell miR-21 expression in dnTGF-β RII mice is also increased compared to control mice. The TGF-β pathway regulates an array of miRNAs. For instance, decreased TGF-β signaling in T-cells induces their miR-21 expression, typically known to amplify the production of IFN-γ and IL-17A via T-cells. Interestingly, transfection of miR-21 in wild type B6 T-cells induced increased production of IFN-γ and TNF-α, modulating the T-cell phenotype seen in dnTGF-β RII mice [38].

### 2.3. Primary Sclerosing Cholangitis

Primary sclerosing cholangitis is a liver disease of unknown etiology characterized by chronic biliary epithelial injury that leads to destruction of the extra- and intrahepatic bile ducts and biliary fibrosis. PSC is a strong risk factor for the development of cholangiocarcinoma (CCA) [48].

Currently there are no studies that have investigated the impact of miRNAs in the pathogenesis of PSC, or whether a potential role exists for miRNAs as diagnostic markers for this disease.

Li and colleagues investigated the potential use of miRNAs as diagnostic tools for the detection of CCA. As a result, they were one of the first groups to report the miRNA content of extracellular vesicles from bile. The bile samples were obtained from patients with CCA, benign biliary tree obstructions, benign bile leaks, and PSC. Using a panel of miRNAs, they were able to distinguish between patients with CCA and PSC with a sensitivity and specificity of 67% and 96%, respectively [49].

Another study investigated the role of miR-7 in cholangiocyte proliferation in the context of bile duct injury in the 3,5-diethoxycarbonyl-1,4-dihydrocoline (DDC) mouse model of sclerosing cholangitis. In this model, wild type mice fed with DDC developed a disease similar to human sclerosing cholangitis. Neurogenin-3 (Ngn-3), which is required for bile duct formation during embryogenesis, is upregulated in DDC mice. Marzioni and colleagues demonstrated that in DDC mice, Ngn-3 controls cholangiocyte replication and collagen secretion in a miR-7 dependent mechanism. Importantly, they also showed that Ngn-3 is overexpressed in bile ducts of patients with PSC [39].

### 2.4. Polycystic Liver Disease

Polycystic liver disease (PLD) is a group of uncommon human disorders that result from structural changes in the biliary tree development. This disorder can occur in isolation, *i.e.*, Autosomal Dominant Polycystic Liver Disease—ADPLD, or more commonly, with an associated renal cystic component, *i.e.*, Autosomal Dominant Polycystic Kidney Disease (ADPKD). Autosomal recessive polycystic kidney disease (ARPKD) is characterized by the formation of multiple cysts in the kidney, liver, and pancreas, and by various vascular abnormalities. Its incidence is 1:20,000 to 1:40,000 [50]. ADPKD occurs in 1:400 patients and results in a slow, gradual, and massive enlargement of the kidneys and ultimately kidney failure. This occurs in the majority of patients by the fifth or sixth decade [51,52]. More than 85% of ADPKD cases are caused by mutations in the *PKD1* gene, with primarily all remaining cases being associated with *PKD2* gene mutations. These genes encode membrane glycoproteins polycistin-1 and 2 (PC1 and PC2). ARPKD is caused by mutations of the polycystic kidney and hepatic disease 1 gene (PKHD1), which encodes the protein fibrocystin/polyductin [53]. ADPLD is a less common disease with the exclusion of renal involvement but otherwise sharing many clinical features of ADPKD, resulting from mutations in either *SEC63* or *PRKCSH* [39]. The majority of proteins encoded by those genes are located in the primary cilia of cholangiocytes and other epithelia [50].

Although the mechanisms involved in the pathogenesis of liver cysts in PLDs are unclear, they are believed to be a result of the dysfunction of several pathways including those of cell proliferation, apoptosis, cell polarity, and fluid secretion. In addition, several studies have demonstrated changes in miRNA patterns present in both cystic cholangiocytes and renal epithelial cells suggesting a novel regulatory mechanism of cystic progression [40,54,55,56].

Previously, our group demonstrated that Cdc25A protein expression, which plays an essential role in cell-cycle progression, is overexpressed in the PCK rats, an animal model of ARPKD, as well as in patients with cystic liver diseases despite no difference in Cdc25A mRNA level. Similarly, we found that miR-15a is down-regulated in cystic cholangiocytes compared to normal cholangiocytes. The fact that miR-15a can bind to Cdc25A mRNA suggests the presence of a posttranslational mechanisms for the regulation of protein expression [55].

Patel *et al.* [57] demonstrated that the miR-17~92 cluster acts as a regulator for kidney cyst growth by increasing proliferation and inhibition of *PKD* genes. *PKD1* and *PKD2* contain conserved binding sites for members of the miR-17 family. One member of the miR-200 family, miR-200b/c/429 represses the expression of *Pkd1* through interaction with these binding sites [58]. Another group, Dweep *et al.* identified, using microarrays, changes in the miRNA expression patterns in PKD/mhm (cy/+), a model of ADPKD [59]. Those miRNAs may have a role in specific pathways underlying PKD [56,60].

Using *in silico* analysis, we examined miRNA signatures in normal and PCK rats. From this, we demonstrated that miR-1, -17, -20, -23, -31, -106, -130, -150, -194, -218, and -342 are predicted to target the mRNAs of the genes mutated in PLD. All of these miRNAs are aberrantly expressed in cystic cholangiocytes and may function in concert with each other affecting critical cellular functions and thus influencing liver cyst growth; 29% of miRNAs target genes related to the cAMP signaling pathway; 5% to the intracellular calcium signaling, 29% to the cell cycle progression; and 12% to fluid secretion [40,56].

Cyst development is driven by somatic second hit mutations, which affects wild type alleles of biliary type cells during early hepatic organogenesis. In PCLD it is thought that subsets of cells behave abnormally during maturation of the ductal plate [61]. MiR-30a plays an important role in ductal plate formation which may promote excess of *EGFR* (the epidermal growth factor receptor). *EGFR* is expressed in the ductal plate and downregulated in mature cholangiocytes [32]. In human cystic cholangiocytes, the level of miR-30a is decreased [40].

Recently, Sun *et al.* have reported that miR-199a-5p is up-regulated both *in vivo* and *in vitro* and may promote cell proliferation through suppression of CDKN1C, which plays an important role in cell cycle regulation [41].

### 2.5. Cholangiocarcinoma

Cholangiocarcinoma is a primary malignancy of the bile ducts [62], and has been increasing in both incidence and prevalence worldwide in recent decades [63]. CCA has poor sensitivity to traditional chemo- and radiation therapy, permitting surgical resection as the only treatment option with curative potential. Some miRNAs can contribute to CCA tumorigenesis by acting as oncogenes or through inhibition of tumor suppressor genes. In addition, deregulated expression of miRNA in malignant cells may contribute to various biological processes [64,65].

Modulation of cell proliferation is an established mechanism through which miRNAs can contribute to tumor growth. Several studies have reported that miR-let-7a, miR-21, -26a, -34a, -421, and -494 are involved in these processes [66,67,68]. Olaru *et al.* found that miR-494 was down-regulated in human CCA samples and its up-regulation induced a decrease in cancer cell growth through multiples targets involved in G1-S (gap 1 to synthesis) transition [42]. Likewise, Yamanaka *et al.* reported the G2/M (gap 2 to mitosis) arrest is induced by miR-494. In particular, the protein levels of six genes are modulated by miR-494: Polo-like Kinase 1 (PLK1), pituitary tumor-transforming gene 1 (PTTG1), Cyclin B1 (CCNB1), cell-division cycle 2 (CDC2), cell-division cycle 20 (CDC20) and topoisomerase II alpha (TOP2A) [69]. Moreover, miR-21, which is overexpressed in CCA, induces posttranscriptional inhibition of both PDCD4 (Programmed Cell Death 4) and 15-PGDH (15-hydroxyprostrangaldin dehydrogenase) and consequently increases the rate of tumor growth [43,44]. Another miRNA overexpressed in CCA is miR-31 which inhibits the protein expression of RAS p21 GTPase-activating protein 1 (RASA1). This promotes proliferation and prevents apoptosis of the cell [70]. Increased levels of miR-25 have shown anti-apoptotic properties by repressing the Death Receptor-4 (DR4) protein expression which protects cells from TRAIL-induced death [71]. miR-144 is down-regulated in CCA both *in vivo* and *in vitro*, and its overexpression is able to attenuate cell proliferation, migration, and invasion. This occurs through the inhibition of AKT and its direct target: LIS1 (platelet-activating factor acetylhydrolase 1b) [72]. Additionally, down-regulation of miR-138 enhances the proliferation, migration, and invasion of cholangiocarcinoma cells. The mRNA of the Ras homolog gene family, member C (RhoC), was identified as a direct target of miR-138.

Based on an established association between chronic inflammation and malignancy, there have been studies investigating the role of miRNAs in IL-6-mediated tumor survival. One group that has studied the relationship between IL-6 and miRNA is Meng *et al*. They assessed the effects of IL-6 on miRNA expression and the subsequent downstream effects of these miRNAs. They demonstrated that IL-6 is able to alter the expression of miRNAs such as miR-let-7a. This particular miRNA contributes to the increased phosphorylation of STAT-3 leading to cell survival [73]. Additionally, the expression of DNA methyltransferase 1 (DNMT-1) can be increased by IL-6. The miRNAs miR-148a, miR-152, and miR-301 have a sequence which is complementary to the 3′-UTR of DNMT-1. The authors found that DNMT-1 was a target of miR-148a and miR-152 using luciferase reporter constructs. The expression of these miRNAs is decreased in CCA cells. Moreover, they noted that precursors of these miRNAs decreased DNMT-1 protein expression, and increased Rassf1a and p16INK4a expression which resulted in a reduction of cell proliferation [45].

An additional process which may be affected by miRNAs is migration which is a phenotypic characteristic of cells required for metastasis. An example is the down-regulation of miR-376c in HuCCT1 cells. This can subsequently regulate EGF-dependent cell migration through repression of *GRB2* translation [74].

The regulation of angiogenesis may be yet another mechanism involved in the modulation of tumor cell proliferation. Indeed, it has been demonstrated that miR-101 inhibits CCA angiogenesis by targeting the 3′UTR of VEGF (vascular endothelial growth factor) mRNA. This can occur either directly or indirectly via inhibition of COX2-derived PGE_2_ signaling [75].

CCA is very resistant to common chemotherapies, like Gemcitabine (Gem). Okamoto *et al.* analyzed miRNA expression in HuH28, a CCA Gem-resistant cell line compared to a sensitive cell line (HuCCT1). They found that miR-29b, miR-205, and miR-221 were down-regulated, and that ectopic overexpression of any one of these miRNAs could restore Gem sensitivity to these cells. Using *in silico* analysis, PIK and MMP-2 were identified as potential targets of these miRNAs [76]. MiR-200b, a highly overexpressed miRNA in malignant cholangiocytes, is involved in cell growth, cell differentiation, and oncogenic transformation by targeting the protein tyrosine phosphatase non-receptor type 12. Furthermore, miR-200b is believed to contribute to CCA chemoresistance by modulating the chemotherapy-induced apoptosis [77].

## 3. MiRNAs as Potential Biomarkers and Therapeutic Targets in the Cholangiopathies

### 3.1. Circulating MicroRNAs

As described above, miRNAs have been identified to be important in the pathophysiology of the cholangiopathies by affecting cell proliferation, apoptosis, migration, inflammation, and chemoresistance of cholangiocytes. In addition, several aspects of miRNAs make them useful for clinical diagnostics. In particular, circulating miRNAs are emerging as potential biomarkers in disease, including most recently, the cholangiopathies.

Exosomes, nano-sized (0.040–0.100 µm) membrane-enclosed vesicles, are a subpopulation of extracellular vesicles (EVs) [78] and have been identified to be potential miRNA shuttles to mediate intercellular communication between cells [79]. EVs are generated within the cytoplasm via inward budding of early endosomal membranes producing intraluminal vesicles (ILVs) [80]. Upon generation of ILVs within the endosome, a multivesicular body (MVB) is formed [80]. Exosomes are released from the cell via endosomal trafficking of multivesicular bodies (MVBs) subsequently followed by MVB fusion to the plasma membrane and exocytic release of the ILVs, now notably referred to as exosomes [80]. Recent studies describe multiple pathways, e.g., ESCRT (endosomal sorting complex required for transport)-dependent [81] and ESCRT-independent [82] (tetraspanins, e.g., CD63) mechanisms, for exosome generation and cellular secretion. Combined interest in exosomes and miRNA targets for diagnostic biomarkers and therapeutic targets has led to several studies analyzing specific miRNA molecules encapsulated within these lipid-enclosed vesicles. More specifically, research has shown specific upregulation and down-regulation of exosomal miRNA and their specific impact on the pathology of certain diseases following target cell uptake [83]. Although, miRNAs have been extensively studied from peripheral blood and serum-free cell culture samples, these miRNAs can often be indiscriminate between diseased and normal patients and susceptible to RNase degradation [83], further supporting increased efforts to isolate enriched miRNA populations packaged as exosomal cargo.

Exosome-induced target cell effects have peaked interest in the significance of exosomal cargo. Functional exosomal miRNA extracted and purified from isolated exosomes via serum and cell culture systems, have been profiled from a variety of cells to study their potential impact on target cells [84,85,86]. Exosomal membrane encapsulation of these small non-coding RNAs is protected and evades degradation from RNases present in circulation, facilitating effective intercellular transfer [87]. The mechanisms of miRNA packaging within exosomes is an area of active investigation; current studies suggest that this packaging may be dependent on the endosomal uptake of the RNA-induced silencing complex [88,89].

Recent data support the concept that exosomal miRNA from a donor cell may induce target cell effects by modifying gene expression of target cells in normal and diseased states. One example discusses miRNA uptake via exosomes derived from embryonic stem cells that can induce alterations in gene expression in target embryonic fibroblasts [90]. In addition, further studies show how uninfected target cells became infected via exosomal miRNA following internalization of exosomes derived from Epstein-Barr virus cells [91]. Other data specifically show how active miRNA packaged within glioblastoma-derived exosomes induced gene modifications in target endothelial cells [86]. Further data show exosomes derived from human colorectal cell lines contain miRNAs known to be involved with colorectal cancer (CRC) metastasis [92], e.g., miR-21, miR-192, and miR-221, further confirmed by RT-PCR [93]. More studies have also reported functional exosomal miRNA following target cell interaction, suggesting their significant role in intercellular communication and disease pathology.

Exosomal miRNA transfer into target cells has also been explored in liver diseases. In HCC, which is the third leading cause of cancer-related deaths in the world [94], miR-21 was detected from exosomes in patient serum [95]. Due to enriched miRNA levels present in exosomes in contrast to whole serum, elevated levels of exosomal miR-21 were observed in patients with HCC compared to patients diagnosed with chronic hepatitis B (CHB) or healthy volunteers. Interestingly, functional clinical tests assessed how elevated levels of miR-21 present in serum of HCC patients directly correlated to cirrhosis and tumorigenesis [95]. More specifically, a positive correlation was demonstrated between high exosomal miR-21 expression and clinical-pathological elements monitored in HCC patients, e.g., liver cirrhosis and tumor stage. Current studies have also observed the specific transfer of exosomal miRNA into a human liver carcinoma cell line, HepG2. These observations were generated through confocal microscopy via tracking exosomal tetraspanin markers, e.g., CD63, CD9, and CD81 [93]. To the best of our knowledge, a cholangiocyte-specific miRNA has not yet been identified from cholangiocyte-derived exosomes; however, these studies are currently being pursued. Taken together, these data further suggest the intercellular communication directed by exosomal miRNAs between cells to not only influence target cellular behavior but also liver disease pathogenesis.

Intercellular transfer of circulating miRNAs via exosome shuttles can be conceptualized to occur in several ways [87]. Exosomes may bind to receptors exposed on the surface of target cells, inducing a signaling cascade and further altering changes in cellular behavior [96,97]. In contrast, exosomes may directly fuse to the plasma membrane via membrane protein complexes followed by cytoplasmic internalization [98]. Further studies also describe endocytic uptake of exosomes mediated by a diverse range of mechanisms, e.g., clathrin-dependent, caveolin-dependent, and micropinocytosis [99]. Specific to the latter targeting mechanism described above, exosomal content, including miRNA, is released into the cell where it can alter gene expression and cellular function; thus, intercellular communication via circulating miRNAs packaged within exosomes may have a distinct role in liver disease pathogenesis.

### 3.2. MiRNAs as Biomarkers for the Cholangiopathies

#### 3.2.1. Cholangiocarcinoma

Cholangiocarcinoma is the only cholangiopathy for which miRNAs in exosomes have already shown potential value as a diagnostic marker (Table 2). Soluble miRNAs extracted from whole bile are rapidly degraded. This has prompted efforts for isolation of human bile EVs to enhance miRNA stability in biliary EVs. In an attempt to develop a novel miRNA panel of specific markers for CCA, miRNAs were extracted from human biliary EVs [49]. Li *et al.* focused on identifying and analyzing an expansive pool of CCA-based miRNAs present in human bile [49]. Assessing the propensity of highly expressed miRNAs to predict CCA diagnosis, a mathematical algorithm was utilized and chosen based on sensitivity and specificity. These studies are critical in showing the potential roles and impact of EV-packaged miRNA and their impact on improving CCA diagnosis.

Shigehara *et al.* have identified 10 miRNAs (miR-9, -145, -105, -147b, -302c, -199a-3p, -222, -942, -let-7f-2, and let7i) in human bile whose levels differ between malignant and benign conditions. After several analyses, they concluded that miR-9 has the potential to be used as a diagnostic indicator for biliary tract cancers [100]. Levels of miR-192 were also reported to be significantly higher in the serum of CCA patients than that of healthy subjects [101]. In addition, the correlation of serum miR-192 with clinicopathological features of CCA showed an association between tumor metastasis and poor survival. When evaluated as a prognostic indicator for CCA, this miRNA demonstrated a sensitivity of 74% and a specificity of 72%. Karakatsanis *et al.* analyzed tissue samples from 60 patients with primary HCC, 21 patients with primary intrahepatic cholangiocarcinoma (ICC) and 98 healthy controls. They found that miR-21, -31, and -223 levels were increased in patients with ICC while miR-122, -145, -200c, -221 and -222 were down-regulated in patients with ICC compared to normal tissues. However, no correlation with clinicopathological features was found [102]. In a retrospective study, McNally *et al.* found 17 upregulated and 26 down-regulated miRNAs in CCA tissue compared to normal bile duct epithelium. The overexpression of miR-151-3p and the down-regulation of miR-126 were identified as potential prognostic markers for CCA progression. The best overall correlation with survival was demonstrated when they were concomitantly dysregulated (58.7 months *vs.* 15.1 months) [103]. Similarly, the overexpression of miR-21 showed a potential role in cancer prognosis. MiR-21 expression was higher in tumors with (1) lower tumor differentiation degree; (2) lymph node metastasis and (3) perineural invasion. All of which are associated with poor survival of CCA patients [104]. The prognostic value of these miRNAs therefore may be important for the stratification of patients for clinical trials as well as identifying those that might benefit from adjuvant therapies.

**Table 2:** miRNAs and their potential as biomarkers of cholangiopathies.

Cholangiopathy	Source	miRNA	Clinical Correlation	Human	Reference
**CCA**					
	**1.** Bile samples from patients who underwent prognostic and/or therapeutic bile drainage	**Sensitivity level of 88.9%** miR-9 miR-302c miR-199a-3p miR-222 **Largest Area under ROC-curve:** miR-9 and miR-145 **Most promising miR:** miR-9	Diagnosis	Bile samples from patients with CCA (cholangiocarcinoma *n* = 7, gall bladder cancer *n* = 2) Bile samples from patients with choledocholithiasis without malignancy or inflammatory condition (age-matched) (*n* = 9)	[100]
	**2.** Surgical specimens of ICC (chemo and radiotherapy naïve)	**Overexpressed in ICC compared to control:** miR-21 miR-31 miR-223 **Down-regulated in ICC compared to control:** miR-122 miR-145 miR-146a miR-200c miR-221 miR-222	No correlation found with clinicopathological features but encouraged further prospective studies to explore the significance of findings	Surgical specimens of ICC (*n* = 21) Liver specimens of healthy controls (*n* = 98)	[102]
	**3.** Surgical specimens of CCA (chemo and radiotherapy naïve)	**Higher expression correlated with worse prognosis:** miR-21	Prognosis	Surgical specimens of CCA (*n* = 41)	[104]
	**4.** Surgical specimens of CCA	**Concordant dysregulation:** miR-151-3p miR-126	Prognosis and potential therapeutic targets	Surgical specimens of CCA with adjacent uninvolved bile duct epithelium (*n* = 32)	[103]
	**5.** Bile samples obtained during ERCP or PTC	**miR-species panel:** miR-191 miR-486-3p miR-1274b miR-16 miR-484	Diagnosis	Bile samples obtained during ERCP from CCA patients (*n* = 46) Controls (*n* = 50)	[49]
	**6.** Tumor tissue from liver fluke (Opisthorchis Vierrini) (Ov)—associated cholangiocarcinoma and serum	**miR significantly higher in the serum of CCA patients compared to healthy subjects:** miR-192	Prognosis and diagnosis	Tumor with adjacent non-tumor tissues (*n* = 30) Sera from patients with ICC (n=51) Sera from healthy subjects negative for Ov (*n* = 32) Sera from subjects infected with Ov (*n* = 10) Subjects with periductal fibrosis (*n* = 20)	[101]
	**7.** Plasma from patients with Ov induced ICC	**Detected in ICC but not control:** miR-483-5p miR-505-3p miR-874 miR-885-5p miR-320b miR-92b-3p miR-1275 miR-1307-3p	Diagnosis	Plasma samples of Ov-induced ICC from patients: Well differentiated ICC (*n* = 4) Moderately differentiated ICC (*n* = 2) Papillary ICC (*n* = 6) Plasma controls (*n* = 5)	[105]
**PLD**					
	**1.** Urine specimens of ADPKD patients	**Higher in urine cells from ADPKD compared to other chronic kidney disease (CKD) patients:** miR-143(2) **Lower in urine cells from APKD compared to other CKD patients:** miR-133b(2) miR-1(4) **Increased abundance in ADPKD urine cells:** miR-223(1) miR-199a(3) miR-199b(1) **Less abundant in ADPKD urine microvesicles compared to other CKD patients** miR-1(2) miR-133a(2)	Monitoring disease progression and treatment response	Urine specimens of ADPKD patients (*n* = 20) Urine specimens of patients with CKD of other etiologies (*n* = 20)	[106]
**PBC**					
	**1.** Serum from patients with PBC	**Downregulated in PBC:** hsa-miR-505-3p miR-197-3p	Diagnosis	Sera of patients with PBC (*n* = 10) (treatment naïve), sera of patients with CH-B (*n* = 5), sera of patients with CH-C (*n* = 5), and sera of healthy controls (*n* = 5).	[107]
	**2.** Peripheral blood mononuclear cells (PBMCs) from PBC patients	**Upregulated in PBC:** miR-15a-5p miR-20a-5p miR-140-3p miR-106b-5p **Down-regulated in PBC:** miR-3654 miR-181a-5p	Diagnosis or treatment	PBC patients (*n* = 9) and healthy controls (*n* = 9) matched by gender and age	[108]
	**3.** Serum from patients with PBC	**Upregulated in PBC:** hsa-miR-122-5p hsa-miR-34a-5p hsa-miR-141-3p hsa-miR-27b-3p **Downregulated in PBC:** hsa-miR-26b-5p	Diagnosis	Sera of patients with PBC (*n* = 207) Sera of healthy controls (*n* = 173)	[109]
**BA**					
	**1.** Serum	**Upregulated in BA:** miR-200b/429	Diagnosis		[110]

ICC: Intrahepatic Cholangiocarcinoma; ERCP: Endoscopic Retrograde Cholangiopancreatography; PTC: Percutaneous Transhepatic Cholangiography; OV: Opisthorchis Vierrini; PLD: Polycystic Liver Disease; ADPKD: Autosomal dominant polycystic kidney disease; PBC: Primary biliary cirrhosis; CH-B: chronic hepatitis B; CH-C: chronic hepatitis C; BA: Biliary atresia.

Recently, Plieskatt *et al.* analyzed tumor tissue samples and matched plasma samples from patients with Ov-induced ICC and controls. They found that adjacent-non-tumor liver tissue obtained from ICC-patients had miRNA expression profiles more similar to the tumor than to control normal liver. In addition, they identified 8 miRNAs which were present in plasma from ICC but not from controls, regardless of histology. Hence, they proposed that such a miRNA profile may have a role as a diagnostic biomarker for this select group of patients and encouraged further studies to explore this potential [105].

#### 3.2.2. MiRNAs as Biomarkers in Other Cholangiopathies

Recently, the utility of miRNAs as a primary diagnostic tool for PBC, PSC, PLD, and other cholangiopathies has begun to be explored. Zahm *et al.* analyzed serum from patients with BA and cholestatic controls matched on age and gender [110]. They identified the presence of increased levels of the miR-200b/429 cluster in BA serum. The same group validated this finding using 24 additional samples from each group. Using this miRNA cluster, they were able to correctly identify 85% of BA patients from the rest of the cholestatic cohort with a sensitivity and specificity ranging between 71%–92%. Using their mouse model of BA, Zahm *et al.* identified a 29-fold increase of miR-200b/429 in EHBDs compared to levels in liver tissue from BA mice. These findings would suggest that EHBDs are the source of miR-200b/429 overexpression. In contrast to other neonatal cholestatic diseases, EHBD injury is a unique feature of BA. As a result, the miR-200b/429 cluster may have a role as a potential biomarker in the diagnosis of BA [110].

## 4. Conclusions

In this review, we have summarized information that is important for understanding the role of miRNAs in the cholangiopathies. We reviewed the mechanisms of potential genetic alterations regulated by miRNAs and the feasibility of utilizing miRNAs as early diagnostic and prognostic biomarkers as well as potential therapeutic targets in the cholangiopathies. MiRNAs not only play a significant role in regulating various processes in normal cells, such as cell proliferation, differentiation, and apoptosis, but also in functioning as oncogenes and tumor suppressors, which can influence chemoresistance. Indeed, the data summarized here justifies continued investigation, including replication of previous findings in larger cohorts of patients and the standardization of miRNA isolation, purification, and amplification protocols.

While the value of miRNAs as diagnostic tools for PBC, PSC, and polycystic liver disease is limitless, there may also be a role for miRNAs in clarifying the pathogenesis of these conditions and in forecasting their progression. Furthermore, miRNAs have great potential value in understanding CCA development and progression as well as serving as potential biomarkers for early detection.

Indeed, the isolation and characterization of miRNAs in biological fluids (e.g., serum, bile, urine, *etc.*) has the potential to change the landscape of patient care, providing a noninvasive and potentially accurate method to identify molecular signatures for early diagnosis and prognosis.

As there have been recent studies suggesting a role for microRNAs in the pathogenesis of the cholangiopathies, particularly in the activation of HSCs, it is imperative to elucidate the specific contribution that cholangiocyte miRNAs have in the development of biliary fibrosis. The involvement of miRNAs in cholangiocyte injury and, consequently, the induction of cholestasis has not yet been revealed.

Furthermore, extensive literature assessed in this review indicates that aberrant expression of various microRNAs is closely associated with chemoresistance of CCA cell lines. It would be interesting to investigate key mediators that regulate the miRNA expression in this setting and in the pathogenesis of other cholangiopathies described herein.
